# Design of a Health Education Program to Manage Chronic Neck Pain: Protocol for a Development Study

**DOI:** 10.2196/56632

**Published:** 2024-10-01

**Authors:** Milagros Pérez-Muñoz, Isabel Rodríguez-Costa, Gerard Lebrijo-Pérez, Daniel Pecos-Martín, Tomás Gallego-Izquierdo, Yolanda Pérez-Martín

**Affiliations:** 1 Physical Therapy Department University of Alcalá Alcalá de Henares Spain; 2 Francisco Díaz Health Center Alcalá de Henares Spain

**Keywords:** neck pain, chronic pain, physiotherapy, health education, emotional expression, biopsychosocial model

## Abstract

**Background:**

Chronic neck pain (CNP) needs attention to its physical, cognitive, and social dimensions.

**Objective:**

We aimed to design a health education program (HEP) with a biopsychosocial approach for patients with CNP.

**Methods:**

A literature search on CNP, health education, and biopsychosocial models was carried out. Seven physiotherapists with expertise in HEPs and chronic pain participated in three teams that evaluated the literature and prepared a synthesis document in relation to the three target topics. Experts compiled the information obtained and prepared a proposal for an HEP with a biopsychosocial approach aimed at patients with CNP. This proposal was tested in the physiotherapy units of primary care health centers belonging to the East Assistance Directorate of Madrid, and suggestions were included in the final program.

**Results:**

The HEP for CNP with a biopsychosocial approach consists of 5 educational sessions lasting between 90 and 120 minutes, carried out every other day. Cognitive, emotional, and physical dimensions were addressed in all sessions, with particular attention to the psychosocial factors associated with people who have CNP.

**Conclusions:**

The proposed HEP with a biopsychosocial approach emphasizes emotional management, especially stress, without neglecting the importance of physical and recreational exercises for the individual’s return to social activities. The objective of this program was to achieve a clinically relevant reduction in perceived pain intensity and functional disability as well as an improvement in quality of life in the short and medium term.

**Trial Registration:**

ClinicalTrials.gov NCT02703506; https://clinicaltrials.gov/study/NCT02703506

**International Registered Report Identifier (IRRID):**

DERR1-10.2196/56632

## Introduction

Neck pain is a frequent reason for medical consultation in primary care [1] and is often a reason for referral to physiotherapy units due to chronic pain [1,2]. It is associated with disability and work absenteeism; it has a 10.4%-21.3% prevalence in the general population. It is more common in women than in men [2,3]. The experience of pain is a significant burden, leading to a deterioration in quality of life that affects both physical and emotional well-being and has an impact on the person’s work, social, and family environments. This results in a high cost for national health systems due to the increased use of health resources, along with important psychological repercussions for those affected, including the risk of associated pathologies, such as anxiety, depression, and sleep disorders. In general, these people report a negative impact on their social relationships: 22% lose their jobs, 4% change jobs, and 27% feel socially isolated and little understood by those around them regarding their condition. Despite the economic impact of the diagnosis and management of these processes, 48% of the individuals are dissatisfied with the long waiting times for treatment, 29% are dissatisfied with the type of treatment received, and a high percentage of health professionals would like to receive additional training to deal with these processes [2,4-6].

Neck pain represents the fourth cause of work incapacity. This musculoskeletal issue is second only to low back pain, with a prevalence of 10% in the population. Treatment techniques and modalities focus on reducing symptomatology through passive or pharmacological interventions [7]. The criteria for approaching these processes by physiotherapists vary according to their training and professional experience. Pain, generally considered as a symptom, is approached with mechanistic methods that tend to produce poor adherence to treatment and often unsatisfactory therapeutic results [8-10]. The National Institute for Health and Care Excellence does not recommend the use of drugs for the treatment of chronic pain, as they have not demonstrated medium- and long-term benefits [11]. It is necessary to seek new approaches based on scientific studies for the treatment of these processes, which guarantee their effectiveness and allow us to know the cost of treatment and the health benefits provided [10,12-17].

Scientific evidence points to the multifactorial nature of chronic pain and the need for educational-therapeutic strategies based on the biopsychosocial model [14,17-19]. The most recent studies show that psychosocial factors influence, favor, and increase the symptomatology and perpetuation of pain. Therefore, it is necessary to understand and manage these factors to provide comprehensive care for people with chronic pain [18,20-23]. Given the importance of physiotherapy in primary care in health promotion and prevention, it is essential to design and develop a strategy for the management of chronic pain. Advances in this field suggest an interdisciplinary intervention, although this is not always possible [24]. Therefore, a physiotherapy program is proposed, based on evidence to facilitate educational-therapeutic interventions [25]. Health education programs (HEPs) provide a valuable tool for physiotherapists to address these processes [26-28]. An HEP should integrate physical techniques that have been shown to be effective, such as therapeutic and recreational exercise; cognitive restructuring techniques; and training in the management of attention, maintained stress, and associated emotions [25-34].

The aim of this work is to design and develop an HEP that considers not only physical, cognitive, and behavioral factors but also other variables that have not been sufficiently taken into account so far, such as emotional factors, values, and beliefs of individuals with chronic neck pain (CNP).

## Methods

### Study Design

In the first phase, 6 primary care physiotherapists with more than 15 years of experience and an expert professor from the University of Alcala were divided into three groups and carried out an exhaustive bibliographic search. This search focused on 3 target topics: CNP, health education, and biopsychosocial model and emotional expression. The search followed a protocol based on the standards of the PRISMA (Preferred Reporting Items for Systematic Reviews and Meta-Analyses) statement [35,36].

The inclusion criteria for the articles were as follows: (1) content focused on at least one of the three target topics; (2) published in the last 10 years; and (3) written in English or Spanish. All papers with their main topic not focused on any of the target topics were excluded.

### Search Strategy

The search was carried out in the Medline databases through the PubMed platform, Cochrane Library, and Physiotherapy Evidence Database (PEDro). The following descriptors included in the Thesaurus were used: “Chronic neck pain,” “Biopsychosocial model,” “Emotional expression,” “Health education,” and “Physiotherapy” using AND/OR as Boolean operators. Studies relevant to the elaboration of the HEP were evaluated. Titles and abstracts were reviewed and a total of 81 articles were selected, of which 64 were excluded because they did not meet the preestablished inclusion criteria, and finally, 23 articles that followed the PRISMA standards were analyzed [12,19,22,24,26,29-31,37-51]. Based on these studies, the educational-therapeutic program was designed.

### Data Analysis

In the second phase, the selected papers were analyzed by 3 expert physiotherapists and 3 professors. Afterward, 3 meetings were held to reach a consensus and select the information collected after testing its methodological quality and to propose an HEP for people with CNP.

The working procedure followed to develop this proposal adhered to the guidelines in the document “Basic methodological recommendations for developing an educational project,” as recommended by the Provincial Directorate of Madrid, which follows the methodology of the Commission for the Validation of Educational Projects of the Community of Madrid for the design of HEPs [35,36].

Once the proposal was developed, it was tested in 3 physiotherapy units. The suggestions for improvement that emerged from 16 patients and 2 physiotherapists were incorporated into the final document, which was evaluated and validated by the Comisión de Validación de Proyectos Educativos de la Comunidad de Madrid (COVAM—Validation Commission of Educational Projects of the Community of Madrid) and is available to all professionals in the library of the community of Madrid [37]. Once the program was developed, a research project was designed to measure its effectiveness.

### Ethical Considerations

This project was approved by both the Clinical Research Ethics Committee of the Hospital Universitario Príncipe de Asturias and the Central Research Commission of Primary Care in Alcalá de Henares (Madrid) (approval number: OE 22/2015). This project was registered in the ClinicalTrials.gov registry (NCT0270350). The beneficiary population of the program included the following: individuals >18 years of age, diagnosed with CNP by their family physician, with physical and psychic capacity to enter the study, and having signed the informed consent. Our goal for the future is to assess the effectiveness of the HEP.

## Results

Following the review of the literature and the consensus of the experts, the program was designed with the following general and specific objectives:

### General Objectives of the Program

- Health: to improve the health of people with CNP and contribute to the improvement of the quality of life of these individuals.
- Educational: to enable people with CNP to understand the factors that modulate the perception of pain and to teach them methods and techniques to help them manage CNP.

### Specific Objectives of the Program

- Cognitive: to know the basis of the neurophysiology of pain, to reconceptualize pain and its origin, to identify the factors that exacerbate pain, to analyze the affective factors that influence pain, to express emotions and feelings identified about pain, and to verbalize the characteristics of pain.
- Emotional: to identify emotions and express them, to share perceived positive and negative experiences, and to become aware of lifestyle changes needed due to having CNP.
- Skills: to perform techniques for managing attention to the body, thoughts, feelings, and emotions, allowing for a release of feelings that can improve health; and to develop skills and learn therapeutic exercises as well as relaxation and visualization techniques.

The HEP consisted of 5 group educational sessions of about 90-120 minutes divided into 2 weekly sessions on alternate days, allowing participants time to perform the tasks at home and be able to integrate them. Each session included an “observer’s guide” designed to collect feedback on areas of improvement. Each session addressed the physical, cognitive, and emotional dimensions of the participant and included the observer’s guidance. Follow-up assessments were conducted 3 and 6 months after the intervention [25].

The number of participants was limited to a maximum of 10. The HEP encompassed cognitive restructuring, therapeutic exercise, attention management, and emotion management. This HEP approach integrated physical techniques that were shown to be effective, such as therapeutic exercise and movement-based play activities. It also included cognitive restructuring techniques, attention management training, and strategies to manage stress and associated emotions through playful movement-based activities [25-34]. The program structure can be seen in Table 1.

**Table 1:** Health education program (sessions, objectives, techniques, and duration).

Sessions (duration) and objectives		Techniques
**Session 1 (120 minutes)**		
	Presenting the program Expressing the patient´s experience of pain Understanding the factors involved in the perpetuation of pain	Talk-colloquium Brainstorming Talk-colloquium
	Becoming aware of and connecting with the body	Visualization technique
	Develop skills for cervical stretching practice	Motor imagery techniques demonstration with training
	Reinforce knowledge, attitudes, and skills learned through work at home	Task diary
**Session 2 (90 minutes)**		
	Clarify doubts and reinforce learning from the first session.	Group discussion
	To understand the importance of dialogue with the body and its physical attitude	Training
	To manage stress, thoughts, and emotions that influence the perpetuation of pain	Demonstration with training (breathing and movement) Relaxation and meditation
	Identify the importance of exercise Initiate gradual exposure to exercise through play and recreational activities	Talk-colloquium Demonstration with training
	Reinforce knowledge, attitudes and skills learned through work at home	Task diary
**Session 3 (100 minutes)**		
	Express and clarify doubts	Talk-colloquium
	Acquire skills to identify thoughts, beliefs, and emotions associated with the perception of pain	Belief restructuring
	Reinforce learned stretches and exercises Playful activities through movement	Demonstration with training Motor imagery techniques (if required)
	Train positive self-communication at home	Mirror technique
**Session 4 (90 minutes)**		
	Clarify doubts and express experiences	Talk-colloquium
	To develop the ability to manage thoughts and emotions involved in physical pain	Belief restructuring Creative resolution technique Anchors
	Reinforce the stress management techniques and the performance of exercises at home.	Task diary
**Session 5 (120 minutes)**		
	Review and reinforce the tasks for the home and clarify doubts.	Talk-colloquium
	Reinforce the ability to manage the emotional burden of the process being experienced	Relaxation techniques
	Develop the ability to live in health	Visualization technique
	Reinforce exercise skills Playful activities through movement	Training
	To reinforce the performance of exercises in the home	Task diary
	Express doubts and ideas about what have been learned	Talk-colloquium
	Evaluate the intervention	Questionnaires

### Session 1

Session 1 was developed in a “large group” format with a maximum of 10 participants. It began with a brief presentation explaining the program, including its objectives and structure. The session aimed to help participants understand the causes of their pain, increase their awareness and connection with their bodies, and develop skills for practicing cervical stretching.

Learning was focused on acquiring skills to de-emotionalize the pain process (fear of movement and catastrophizing). Techniques were used to help the connection with the body and develop skills for practicing stretching and cervical exercises. Initially, motor imagery techniques were used until stretching could be initiated with real movements.

Finally, the participant was asked to practice and write down the tasks learned and review these tasks at home, and the program materials were handed out.

### Session 2

Session 2 began with the clarification of doubts and reinforcement of the learning from session 1, with an emphasis on the importance of dialogue with one’s own body. We worked on the ability to manage stress, thoughts, and emotions, teaching participants to consciously change their responses through body awareness and movement. It also highlighted the importance of gradual exposure to physical exercise and learning how to stretch and do basic cervical spine exercises. Playful aerobic activities were to promote social participation and attention management. It was explained how to do the exercises collected in the task diary at home.

### Session 3

We began by dedicating a few minutes at the beginning of the session to clarify doubts about what was covered in previous sessions. This session delved into the identification of emotional impacts as possible causes of the perpetuation of neck pain, with special emphasis on social pain and interpersonal relationships.

It was also recommended that the participant took note of the moment when the pain appeared, the emotional situation prior to the onset, and the context surrounding the participant to analyze and explore the emotion-situation-pain relationship.

The session included a review and reinforcement of the stretching and cervical exercises learned in previous sessions, along with playful activities through body movement. For home practice, participants were instructed to continue with the same exercises as in session 2. Additionally, an exercise focused on training positive self-communication was introduced in this session.

### Session 4

As in previous sessions, participants were invited to express their doubts and experiences about their neck pain.

Practices were done to acquire skills to learn to manage limiting thoughts and beliefs and include strategies to change them. The techniques learned to manage stress were reinforced, and playful activities were carried out through body movement and therapeutic exercise.

Practicing the techniques used to manage stress as well as stretching and cervical exercises were requested to be done at home.

### Session 5

As in previous sessions, participants were invited to express their doubts and experiences about their neck pain.

The learning focused on the acquisition of skills to reduce the emotional burden of pain and improve health and well-being. Additionally, we reviewed and reinforced exercises to be performed daily at home, and the dates of their execution were noted down until the review or follow-up performed at 3 and 6 months, respectively, from the end of the HEP.

To ensure adherence to the HEP, participants were informed that a phone call would be made during the first 3-month follow-up and another one would be made during the second follow-up to clarify any doubts and encourage them to continue performing the tasks.

## Discussion

The role of the physiotherapist includes facing important challenges due to new strategies that have emerged in response to the health needs of people. The HEP programs are a fundamental tool in primary care, to be used by these professionals who play a very important role in influencing and promoting behavior change related to the lifestyles of people with chronic pain. Their activities should not only provide assistance but also promote health and prevent issues. The primary areas of application are musculoskeletal (69%) and therapeutic physical activity (20.6%) [28,46].

Scientific literature includes interventions for chronic pain that use pain education as a tool by explaining the neurophysiology of pain. These interventions usually yield positive results in the short and medium term, reducing pain intensity and functional disability while improving the quality of life for individuals with chronic pain, compared to conventional physiotherapy interventions [48,52-55].

Other studies have shown that this type of educational strategy can also have a positive short- and medium-term effect on catastrophizing and physical performance [31,48-50,53]. In light of the current literature available, education in pain neurophysiology is considered a necessary but not sufficient pillar of an effective approach. For this reason, interventions have been developed that combine education in pain neurophysiology with therapeutic exercise and other forms of care.

Current scientific evidence points to the effectiveness of combining pain education with physiotherapy interventions based on therapeutic exercise in the short and medium term. This approach has been shown to improve functional disability and reduce fear avoidance [43,53,55-58].

Literature suggests that individuals should be considered as biopsychosocial beings; their beliefs; cognitive, emotional, and behavioral factors; as well as their social context play crucial roles in the manifestation, development, and perpetuation of pain [20,23,59]. Therefore, psychosocial factors must be taken into account in actively addressing these processes.

There are several studies that demonstrate the importance of considering these factors and the awareness of the individual’s thoughts, feelings, and emotions in the face of pain to reduce symptomatology [12,20,23,26,28,46,48,50,53,57,59].

However, although physical therapists can often recognize the influence of these factors, few have developed the skills to successfully assess and manage them. It is essential that physical therapists acquire the knowledge, attitudes, and skills necessary to be able to perform an active and structured approach to physical therapy, focused on the person with chronic pain, and to be able to use scientifically based tools and techniques.

Some programs evaluate the effect of cognitive-behavioral therapy in the improvement of pain, disability, and quality of life of individuals with CNP. Changes were observed without clinical relevance in the long term. This might be due to the fact that although these psychosocial factors are mentioned, they are not specifically addressed in the interventions. Additionally, these interventions often do not take into account the impact of social pain on the perception of physical pain even though both types of pain activate common pathways and brain centers. Social pain, resulting from feelings of social exclusion or rejection in interpersonal conflicts, affects people’s daily lives in general and can intensify the perception of pain in individuals with chronic pain [21]. If social pain is not taken into account and addressed in interventions, achieving clinically relevant results becomes more challenging. Furthermore, there is often a lack of direct approaches to sustained stress or the individual’s internal emotional disturbances [38,60-64].

Patients with chronic pain are known to be subjected to high stress levels. The nervous system’s response to stress interferes with perceived pain [6]. Therefore, individuals with chronic pain must learn to manage stress. The lack of inclusion of tools to address this may partly explain the absence of clinically relevant results in the management of chronic pain. Other interdisciplinary programs show the importance of approaching patient education from a biopsychosocial point of view. Moreover, in all of these programs, the active participation of the patient is considered essential [10,14,25,26,56]. To achieve the objectives, it is necessary for the individuals to take control of their process and become involved in its resolution with the appropriate support of health professionals.

Therefore, an HEP has been proposed that presents strategies with scientific evidence to address psychosocial factors [26,38,45,65-67], such as breathing and relaxation techniques, motor imagination, as well as play and recreational activities through body movement. This program also considers physical factors and promotes the return to normal activity and social participation for individuals with chronic pain, the importance of which is shown by systematic reviews that provide evidence of the benefits of exercise for CNP [39,68-70]. The fact that the program is conducted in small groups also allows the participants to get to know each other, share experiences, and establish social bonds and support. These interactions, both within and outside the program, can facilitate social participation and aid in their reincorporation into social life.

Regarding the number of program sessions and their duration, the literature consulted includes programs ranging from 1 or 2 sessions to some with 11 or more sessions. As for the duration of the sessions, the proposals range from 30 minutes to 4 hours [33,51,71].

Due to this variety in program structures and based on different guides for the elaboration of educational projects, along with the authors’ extensive experience in the field of primary care, the educational proposal of this program is for 5 sessions of 2 hours each—a medium term among all those consulted [72-74]. This duration is considered sufficient to work on both physical and psychosocial factors, without delaying the process excessively, to achieve good adherence and minimize participant dropout. However, as future lines of action, we are considering increasing the number of sessions and their duration to allow for more gradual and in-depth interventions, both in addressing psychosocial factors and incorporating therapeutic exercises of greater intensity and strength, as indicated by current research in this field.

The review, revision, and possibility to raise doubts in each session help secure knowledge, reinforce new attitudes, support the changes that are taking place, and minimize possible and unlikely adverse effects. This approach has proven to be an effective and safe intervention.

We are working on extending the number of sessions and their duration with the aim of focusing deeply on psychosocial factors and incorporating more intense and strength-based therapeutic exercises, as indicated by current research in this field.

Currently, the approach to chronic pain, and in particular CNP, requires attention to the associated biological and psychosocial factors. The HEP program, with a biopsychosocial approach, emphasizes understanding one’s own processes and questioning beliefs about pain through pain education, alongside managing emotions and stress through body movement. In addition, HEP emphasizes the use of therapeutic and recreational exercises for restoring function and promoting social participation in individuals with CNP, both in the medium and long term in individuals with CNP. More scientific research should be devoted to studies with methodologies that effectively define practical methods for individuals to learn how to manage their feelings and emotions as well as identify interventions that account for these factors. In the meantime, all health care professionals should be aware that “invisible psychological factors” play a role in chronic pain processes. These factors influence how individuals perceive and experience their pain and how they respond and behave to this condition, which affects the person as a whole.

## Abbreviations

CNP: chronic neck pain
COVAM: Comisión de Validación de Proyectos Educativos de la Comunidad de Madrid
HEP: health education program
PRISMA: Preferred Reporting Items for Systematic Reviews and Meta-Analyses
